# Standard Urine Collection Bag as an Improvised Bogotá Bag as a Temporary Abdominal Closure Method in an Open Abdomen in Preventing Abdominal Compartment Syndrome

**DOI:** 10.1155/2021/6689000

**Published:** 2021-01-29

**Authors:** Ahmed Shabhay, Zarina Shabhay, Kondo Chilonga, David Msuya, Theresia Mwakyembe, Samwel Chugulu

**Affiliations:** ^1^Department of General Surgery, Kilimanjaro Christian Medical University College (KCMUCo), P.O. Box 2240 Moshi, Tanzania; ^2^Department of General Surgery, Kilimanjaro Christian Medical Centre (KCMC), P.O. Box 3010 Moshi, Tanzania; ^3^Institute of Infectious Diseases and Research, Lugalo Military College of Medical Sciences (MCMS) and General Military Hospital (GMH), P.O. Box 60000 Dar es Salaam, Tanzania; ^4^Muhimbili Orthopedic Institute, P.O. Box 65474 Dar es Salaam, Tanzania

## Abstract

Primary abdominal wall closure post laparotomy is not always possible. Certain surgical pathologies such as degloving anterior abdominal wall trauma injuries and peritoneal visceral volume and cavity disproportion render it nearly impossible for the attending surgeon to close the abdomen in the first initial laparotomy. In such surgical clinical scenarios leaving the abdomen open might be lifesaving. Forceful closure might lead to abdominal compartment syndrome and impair respiratory status of the patient. Open abdomen closure techniques have evolved over time from protection of abdominal viscera to complex fascia retraction prevention techniques. Silo bags, i.e., (Bogotá Bags), are relatively cheap, available materials used as a temporary abdominal closure method in limited resources settings. Despite its limitations of not preventing fascia retraction and draining of peritoneal fluid, it protects the abdominal viscera. We report a case of a 29-year-old male who developed incisional anterior abdominal wall wound dehiscence. He was scheduled for emergency explorative laparotomy. Intraoperatively, multiple attempts to reduce grossly dilated edematous bowels into the peritoneal cavity and fascia approximation into the midline were not possible. A urinary collection bag was sutured on the skin edges as a temporary abdominal closure method in prevention of abdominal compartment syndrome. He fared well postoperatively and eventually underwent abdominal incisional wound closure. In emergency abdominal surgeries done in limited surgical material resource settings were primary abdominal closure is not possible at initial laparotomy, sterile urine collection bags as alternatives to the standard Bogota bags as temporary abdominal closure materials can be safely used. These are relatively easily available and can be safely used until definite surgical intervention is achieved with relatively fewer complications.

## 1. Introduction

Despite an open abdomen being accompanied with substantial morbidity and mortality, there are surgical conditions which warrant it as a temporary measure to manage surgical pathologies as life-saving measures [1]. However, the longer the abdomen is left open, the higher the potential for morbidity [2]. Prosthetic materials may be used as the temporarily abdominal closure method [3]. These include improvised plastic silos or sterilized intravenous bags coined Bogotá bags by Mattox [4, 5] while observing Oswaldo Borraez in Bogota, Colombia, stitched to the anterior abdominal wall skin [6–8].

The Bogota bag is a simple plastic material bag which is applied over an anterior abdominal incisional wound and sutured over the skin or fascia edges [9]. It gives room for the peritoneal viscera to expand preventing increased intraabdominal pressure [9]. We report a case of a 29-year-old male who had a urine collection bag applied on his anterior abdominal wall incisional wound as a temporary abdominal closure measure as a means to prevent abdominal compartment syndrome.

## 2. Case Presentation

A 29-year-old male was referred to our center with clinical features suggestive of intestinal obstruction. He had three prior histories of abdominal surgeries two years prior current complaints. On examination, he was ill-looking, in distress, pale, dehydrated, and conscious with the Glasgow coma scale score of 15. His vitals were blood pressure 110/75 mmHg, pulse rate 135 beats/minute, temperature 36.7°C, and oxygen saturation was 94% on room air with random blood glucose of 6.7 mmol/L. His hemoglobin level was 15 g/dL.

He was scheduled for emergency explorative laparotomy. Intraoperative findings were a constriction band 10 cm from the ileocaecal junction with grossly dilated viable small bowels from ligament of Treitz to the obstruction point. Adhesion band was released, and bowel patency was established. Abdominal fascia was closed with vicryl number 1 and skin nylon number 2-0. Postoperatively, the patient progressed well initially. His postoperative albumin level was 16.09 g/L.

However, he developed incisional anterior abdominal wall wound dehiscence day seven postoperatively. He was scheduled for a second emergency explorative laparotomy. Preoperative hemoglobin was 11.9 g/dL. Intraoperative findings were a gapped abdominal fascia, grossly dilated and edematous bowels, and immature adhesions, and small amount of intraperitoneal pus was noted. Appendectomy was done, and thorough abdominal lavage with copius Normal saline infusion, refreshing of abdominal fascia and skin edges, one abdominal drain left in situ, and tension sutures were applied. Postoperatively, he developed generalized body edema, abdomen was distended, and bowel sounds were reduced with limited bowel movement. Serous fluid discharge per incision site was noted. Postoperative serum albumin was 13.12 g/L and total protein 32.8 g/L.

On the seventh day posttension sutures, he developed a second incisional anterior abdominal wall wound dehiscence. He was scheduled for a third emergency explorative laparotomy. Intraoperative findings were remnants of broken-down tension sutures, gross dilation, and edematous bowels from gastrium to ileum together with the large colon up to the sigmoid colon with immature adhesions.

**Figure 1:**
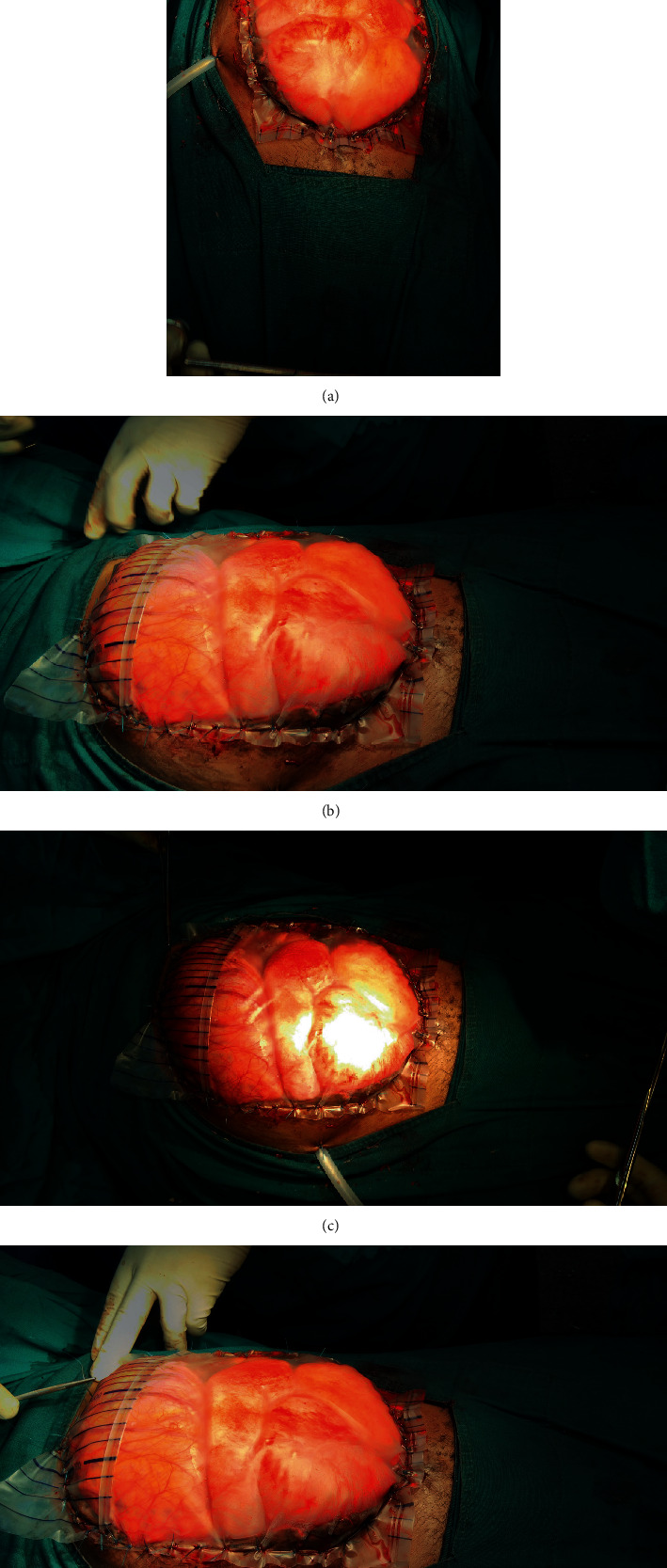
Standard urine collection bag sutured over anterior abdominal wall incisional wound exposing oedematous bowels with abdominal drain in situ.

Abdomen lavage was done, gentle decompression of bowels through the rectum done. Multiple attempts of reduction of bowels into the peritoneal cavity were attempted with approximation of abdominal fascia in the midline without success. A clinical decision was made to abort attempts to forcefully reduce the grossly dilated edematous bowels into the peritoneal cavity in fear of abdominal compartment syndrome and respiratory failure due to cephalad displacement of diaphragm. Resection and primary anastomosis of a segment of ileum to reduce the volume of intraabdominal contents was not an option due to his low serum albumin and total proteins levels. A standard empty sterile double-layered polyvinyl urinary collection bag was applied over the open anterior abdominal wall incisional wound (Figures 1(a)–1(c).

The urine bag was cutand trimmed to fit the open abdominal wall incisional defect. It was stitched to anterior abdominal wall skin edges using interrupted prolene 0 stitches at an interval of 2 cm apart. An intraabdominal drainage tube was placed in situ for drainage of peritoneal fluid.

Postoperatively, the patient edematous bowels could be visible from the urinary bag. His generalized edema subsided and was scheduled for elective tension suture application 7^th^ day post urinary bag application. Intraoperatively, abdominal fascia was slit bilaterally and mobilized, and horizontal mattress sutures were applied with nylon number 1. The skin was closed with horizontal mattress tension sutures. He was discharged and reviewed 5 weeks later. He had on and off episodes of relative constipation which resolved on conservative management. No anterior abdominal wall ventral hernia was noted.

## 3. Discussion

There are a number of techniques from authors for open abdomen/temporary abdominal closure methods; however, there is no documentation of a proven superior technique [1, 10]. In most surgical clinical settings, postlaparotomy primary anterior abdominal wall incisional wound fascial closure is done [5]. However, some surgical clinical settings might warrant an open abdomen as part of damage control surgery or prevention of abdominal compartment syndrome. Literature has disproved the old adage of closing the abdomen at “all cost” [11]. However, no definitive data about the open abdomen technique's epidemiology and outcome is available despite being employed in a number of surgical clinical settings [11]. These include visceral peritoneal cavity size disproportion in organ transplantation procedures, severe trauma, infected pancreatic necrosis, necrotizing infection of anterior abdominal wall tissues, and ischaemic viscera with a second look planned surgery [6]. It should be noted however that there is no substitute for the anterior abdomen wall fascia and skin regarding laparotomy incisional wound closure [12].

In these challenging clinical scenarios, prosthetic materials are used to temporarily close the abdominal wall as forceful primary abdominal fascia closure could produce undue tension on fascia [3]. However, there is no documented ideal prosthesis for temporary abdominal closure [1]. A number of materials have been used by different authors for temporary abdominal closure such as polyglycolic acid mesh (Dexon) and absorbable woven polyglactin mesh (Vicryl) [1]. However, of late, the most popular material preferred by surgeons has been the sterilized, opened irrigation genitourinary bag (Bogotà bag) [1, 5].

It should however be noted that the material of the Bogotà bag used for temporary initial abdominal closure should preferably be durable enough to preserve its integrity and protect intraperitoneal contents, flexible to avoid inflicting trauma to intraperitoneal viscera organs, thus leading to enteroatmospheric fistula, noncarcinogenic, biologically inert not to provoke an inflammatory response cascade and relatively cheap and easily available [6, 8]. In limited resources, clinical settings were the Bogota bag is not available sterile soft intravenous fluids bags, urine collection bags, Esmarch elastic bandages [13], bowel bag, Sterile Vi-Drape, or Silastic cloth that can be used as an alternative [6].

The Bogota bag is the relatively inexpensive temporary abdominal closure method that is currently available with primary closure rates ranging from 12 to 82% and enteroatmospheric fistula development rate ranging from 0 to 14.4% [6]. It is a treasured method and is the favored closure system to prevent or treat abdominal compartment syndrome. It may decrease respiratory and renal impairment related with intraabdominal pressure [6]. It acts as an airtight barrier preventing fluid loss and bowel evisceration [8]. It has an advantage of being transparent providing good exposure of intraperitoneal organs between reexplorations as abdominal contents that can be observed through it [8, 14] . It also prevents fistula formation [14]. The bag is changed weekly or in two weeks if definite fascia closure is still not possible [9]. In most cases, the abdomen is closed within two weeks [9].

With regards to time frame on eventual abdominal closure with respect to the Bogota bag, a retrospective analysis by Hu et al. [4] concluded that the time to abdominal closure was the longest in the cohort managed with the Bogota Bag. This might be due to its inability of not permitting drainage of intraabdominal fluid that accumulates during resuscitation of the patient with accompanying lateral retraction of the skin and fascial edges [4, 6]. In our case, we inserted an abdominal drain in situ to drain peritoneal fluid accumulation.

In a systematic literature review, Ribeiro et al. [15] observed that the Bogota bag technique had the advantage of availability of the materials and low cost. The use of the Bogota bag was reported to be associated with higher rates of skin lacerations and evisceration, a higher need for a peritoneal drain and higher rates of adhesions formations of peritoneal organs with the anterior abdominal wall.

A comparative study on the Bogota bag versus vacuum-assisted closure in Brazil by Rodrigues et al. [5] concluded the vacuum-assisted abdominal closure and the use of the Bogota bag with respect to the outcome on the integrity of the abdominal wall, and the results did not differ significantly; although, in their center, there was a tendency of using the Bogota Bag preferably in traumatic cases and vacuum-assisted closure in nontraumatic cases.

In a prospective observational cohort study by Coccolini et al., with data from the International Register of Open Abdomen (IROA) on techniques of open abdomen closure, the Bogota bag and skin closure techniques seemed to improve results in trauma cases [11]. In the adult population, 31.8% patients had the Bogota bag, and the skin closure technique was applied. The definitive and fascial closure rates were at 83.2% and 71.3%, respectively. The enterocutaneous fistula formation rate was at 7.4% [11]. Overall, the complication rate during treatment was at 35.8%. The average number of days the abdomen was open was 5. In trauma patients, the definitive closure rate was higher at 92.9%. Overall, mortality in the Bogota bag and skin closure cohort was at 17%. Patients with peritonitis, mortality was the highest at 27.5%. However, in trauma patients, mortality was the lowest at 7% compared to other techniques [11]. It should however be noted that the high mortality rates documented might be due to the primary underlying surgical pathology leading to an open abdominal closure and not directly to the Bogota bag method itsel f[8, 12]. Peritonitic patients had lower definite and fascial closure rates at 72.5% and 62.8%, respectively. Complications during treatment were the lowest at 40% compared to other techniques [11].

Setbacks of the Bogota bag include inability to reapproximate the abdominal fascia in the midline for closure due to fascia retraction posttemporary abdominal closure leading to large ventral hernias and loss of the abdominal domain [4–6, 8, 14]. Lateral retraction can be prevented by using of intravenous tubes as sutures [9]. They are tightened gradually as edema subsides thus approximating the fascial edges. Development of an enteroatmospheric fistula may be regarded as the most dreaded and potentially dangerous serious complication [8]. Other documented complications include fluid and protein loss, nutritional deficiency, and catabolic state [8]. Higher fascia closure rates have been detected in vacuum-assisted closure methods as these systems provide constant fascial traction [10].

In our case, the clinical settings faced by the attending surgeon were as follows: the surgery was an emergency procedure, it was not anticipated that a temporary abdominal closure technique would need to be employed, and there was no available standard used temporary closure materials such as vacuum-assisted closure techniques, meshes, or the Wittmann patch. The attending surgeon used his own ingenuity to use a urine collection bag as the only available alternative to the Bogota Bag. This was done as a temporary closure method of the anterior abdominal wall incisional wound after multiple failed attempts to approximate the abdominal fascia to the midline due to grossly dilated edematous bowels. This clinical setting created a surgical dilemma on the way forward in an austere situation. Forceful midline approximation of the abdominal fascia would put the patient at risk of development of abdominal compartment syndrome, or sustain yet another abdominal wall incisional wound dehiscence status.

## 4. Conclusion

The standard urine collection bags can be safely used as alternatives to the Bogotà bag. In limited resource settings and in emergency open abdominal closure methods, urine collection bags are effective, relatively cheap, and useful materials.

## Data Availability

All data and materials pertaining to this case report can be made available on request.
